# Investigating Gaze of Children with ASD in Naturalistic Settings

**DOI:** 10.1371/journal.pone.0044144

**Published:** 2012-09-24

**Authors:** Basilio Noris, Jacqueline Nadel, Mandy Barker, Nouchine Hadjikhani, Aude Billard

**Affiliations:** 1 Learning Algorithms and Systems Laboratory, Ecole Polytéchnique Fédérale de Lausanne, Lausanne, Switzerland; 2 Emotion Centre, Hôpital de La Salpétrière, Paris, France; 3 Lausanne University Department of Child and Adolescent Psychiatry, University Hospital of Canton de Vaud, Lausanne, Switzerland; 4 Brain and Mind Institute, Ecole Polytéchnique Fédérale de Lausanne, Lausanne, Switzerland & Martinos Center for Biomedical Imaging Massachusetts General Hospital/Healthcare Management Systems/HST, Boston, Massachusetts, United States of America; Lyon Neuroscience Research Center, France

## Abstract

**Background:**

Visual behavior is known to be atypical in Autism Spectrum Disorders (ASD). Monitor-based eye-tracking studies have measured several of these atypicalities in individuals with Autism. While atypical behaviors are known to be accentuated during natural interactions, few studies have been made on gaze behavior in natural interactions. In this study we focused on i) whether the findings done in laboratory settings are also visible in a naturalistic interaction; ii) whether new atypical elements appear when studying visual behavior across the whole field of view.

**Methodology/Principal Findings:**

Ten children with ASD and ten typically developing children participated in a dyadic interaction with an experimenter administering items from the Early Social Communication Scale (ESCS). The children wore a novel head-mounted eye-tracker, measuring gaze direction and presence of faces across the child's field of view. The analysis of gaze episodes to faces revealed that children with ASD looked significantly less and for shorter lapses of time at the experimenter. The analysis of gaze patterns across the child's field of view revealed that children with ASD looked downwards and made more extensive use of their lateral field of view when exploring the environment.

**Conclusions/Significance:**

The data gathered in naturalistic settings confirm findings previously obtained only in monitor-based studies. Moreover, the study allowed to observe a generalized strategy of lateral gaze in children with ASD when they were looking at the objects in their environment.

## Introduction

Impairments in social interaction and communication are the main characteristics of Autism Spectrum Disorders (ASD) [1]. The visual manifestations of these impairments have been the focus of many studies, and several atypical viewing strategies have been documented in ASD (for a review, see [2], [3]). While the underlying causes of gaze peculiarities in autism are not clear, and subject to controversy [2], [4], there is evidence for abnormal gaze behavior towards faces in ASD. Atypical visual behavior is most apparent when studying gaze directed towards social stimuli such as faces [5], more so when these appear as dynamic stimuli [6]. Individuals with ASD show a weaker tendency to initiate and maintain eye to eye contact with other people, and give less attention to faces [7], [8]. This is true when the face stimuli are shown as isolated images [9], [10] and is accentuated when faces are presented in a natural social interaction [11], [12]. Individuals with ASD also have a tendency to look more at the mouth than the eyes [9], [11], [13], [14]. Given the importance of eyes as a social cue, this behavior likely explains the reported difficulties for people with ASD in estimating emotions and judging the mental state of others [9], [15]–[17]. The same tendency may also contribute to the reported difficulty in recognizing faces [10], [13], [18], although the results on this issue are controversed [2].

Some studies have directly addressed processing of visual information (for a review, see [3], [19]), and shown difficulties in disengaging from competing stimuli [20], [21], atypical attention shifts [5], [22] and strategies of visual exploration to overcome perception deficits [23]. In this direction, Senju and Johnson [24] hypothesize, on the basis of fMRI evidence, that perceived eye contact (which they term *eye contact effect*) modulates the activation of the social brain network. The atypical pattern of eye contact consistently reported in ASD individuals may allow them to weaken the *eye contact effect* and narrow down the processing of other types of social information provided by the visual scanning of faces [25]. They argue that infants at high risk of autism do not show avoidance of eye contact but present atypical brain responses suggesting atypical top-down modulations of neural activities in response to eye contact.

Many recent studies have focused on a fine partitioning of the face region and studied the gaze towards eyes, eyebrows, mouth and other facial features. Among the most notable, [11] studied the gaze of adults with ASD to eyes, mouths, bodies and objects in videos of social situations. Adults with ASD looked less at the eyes than controls and their gaze was directed more often at the mouth rather than the eyes. In a longitudinal study of at-risk infants, [26] analyzed the gaze towards the face of their mother and did not find a significant correlation between gaze towards the eyes at six months of age and diagnosis of autism. However, they noticed that a high amount of gaze to the mouth at six months was correlated to a higher verbal development later on, underscoring the importance of the role of gaze in speech development. Indeed, the mouth provides a physically contingent relation to speech sounds, and children with ASD may be looking at it to overcome their difficulties in verbal development [27]. In summary, these reports show how studying the gaze of specific features can increase our knowledge of how autism affects the development of children.

The most commonly used techniques to study gaze peculiarities rely on eye-tracking systems, that usually include a device that shows a visual stimulus on a monitor (e.g. Tobii, ISCAN) [7], [11], [12], [14], [18], [28], [29]. Taking a different approach, Scassellati and colleagues [30] monitored the gaze of children with ASD when interacting with a robot face. They used an automated face tracking system on video recorded from a camera mounted on the robot's head. This approach contributed to a better understanding of how children with ASD interact with human-like agents [31]–[33]. However, placing a camera on the head of the interaction partner provides information only when the child looks at the other. To obtain a first-person point of view, Yoshida and Smith [34] used a small head-mounted camera that recorded a wide-angle image of the child's point of view. They were thus able to record the contents of the child's broad field of view, without having to manually estimate the child's head direction from an external camera. A limitation of this setup, however, was that the device did not measure the direction of the eyes. In our studies, we use the WearCam, a device that monitors both the broad field of view and the direction of the gaze, from the viewpoint of the child [35].

As the atypical behavior in children with ASD is more pronounced in natural social settings than in experimental settings with isolated stimuli [6], [36], our study targets the behavior of children taking an active role in a dyadic interaction with an adult. We are specifically interested in monitoring what the child is looking at, both when looking at an adult and when looking elsewhere. The apparatus we use allows us to monitor the child's interactions from a first-person point of view, and thus to study the use of both the central and peripheral vision during the interaction. Our study proposes focuses on the natural interaction between a child and an unknown experimenter in a semi-structured setting, and comprises a subset of the Early Social Communication Scale (ESCS) [37], [38], an instrument designed to assess social development before the development of language, which is used both in clinical assessment of ASD and in research studies on ASD [38]. The ESCS is used on a regular basis as a screening and diagnosis tool in clinical settings in several countries [39].

## Methods

### Participants

We recruited ten children with ASD (9 boys, 1 girl) from the child Psychiatric Departments of the University hospitals of Geneva and Lausanne in Switzerland. Their mean Chronological Age (CA) was 5.3 (1.8) [2.8–8.8] (Values are presented in the form Mean(SD)[Range]). All children had been previously diagnosed with ASD. Their diagnosis was confirmed using the revised ADI-R [40]. They were matched with ten Typically Developing children (TD) on gender and Adaptive Behavior age (ASD: 2.9 (1.7) [1.3–7.1], TD: 2.9 (1.6) [1.3–6.9]). The choice of Adaptive Behavoir, which was assessed using the Vineland Adaptive Behavior Scale [41], was made to ensure that children would have similar skills in everyday and interaction tasks. The details on the ADI-R and Vineland scores of the participants for each sub-scale are presented on Table 1. The CA for the control group was 3.3 (1.9) [1.2–7.1].

**Table 1:** Scores of the ASD and TD children on the ADI and Vineland Adaptive Behavior Scales.

Variable	ASD	TD	p
	ADI-R^a^		
Recipr. Social Inter.	22±4 (14–28)		
Language/Comm verbal	16±4 (11–22)		
Language/Comm non-verbal	11±2 (7–14)		
R,R,S Behaviors	6±3 (2–11)		
	Vineland^b^		
Communication	2.7±2.3	3.0±2.0	0.68
Autonomy	2.8±1.9	2.7±1.8	0.59
Socialization	2.0±1.5	2.7±1.7	0.40
Mobility	3.2±1.3	2.3±1.3	0.15
**Adaptive Behavior** *	**2.9**±**1.7**	**2.9**±**1.6**	**0.90**
Chronological Age	5.3±1.8	3.3±1.9	<0.01

a mean ± stdev (ranges).

b mean ± stdev.

* used to match devel. Age.

Each child took part in one session that lasted a maximum of 10 minutes. All children accepted to wear the device (see description in the next section) and participated successfully in the interaction. As a consequence, no data had to be removed from the experiments.

#### Ethics Statement

All parents gave their written informed consent including permission to use video recordings and pictures of the children for scientific publications. The experimental protocol and consent form was approved by the Ethics Committee of the University Hospitals of Geneva and Canton de Vaud.

### Apparatus

**Figure 1:**
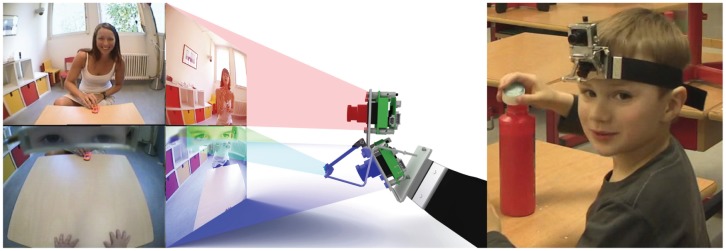
The WearCam device. *Left*: Schematic view of the images recorded by the WearCam, highlighted are the interaction zone (top), the eyes reflected by the eye-mirror (middle) and the manipulation zone (bottom). Software for automatic monitoring of the child's gaze and detection of human faces in the camera images is used to quantify, among other factors, the frequency and length of time during which the child looks at human faces. *Right*: The WearCam worn by a typically developing child.

We recorded the interactions using the WearCam [35], a wearable eye-tracking device (see Figure 1). The device simultaneously records the eyes of the child and an image of the field of view in front of the child, thereby allowing to monitor the direction of gaze and focus of attention. The WearCam weighs approximately 180 g and has a field of view measuring 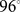 both horizontally and vertically. The visual field of children is considered typical when it extends above 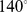 horizontally, and 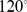 vertically [42], the WearCam therefore captures approximately 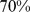 of the effective field of view horizontally, and 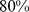 vertically. Simultaneously, the WearCam records an image of the eyes of the child, which are reflected by a small mirror. The image acquisition speed for the cameras was 25 Hz, corresponding to one image every 40 msec, and the recorded image resolution is of 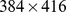 pixels. The acquisition speed of the WearCam does not allow to measure quick saccades, as only events slower than 40msec can be measured with confidence (as two successive image frames are necessary to sense a change), but it can be used to measure typical gaze fixations.

**Figure 2:**
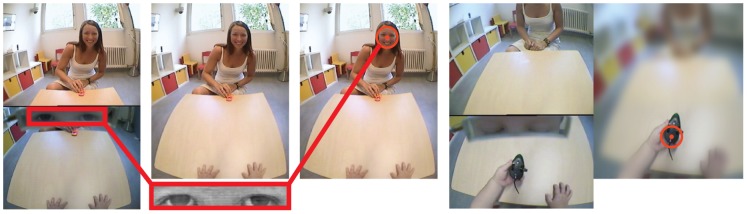
Eye-Tracking process. 1*^st^* column: the location of the eyes in the image is extracted automatically during post-hoc calibration. 2*^nd^* column: the direction of gaze is computed automatically from the eyes image through support vector regression. 3*^rd^* column: to highlight the direction of central vision (indicated by a crosshair), the image is blurred except for an area of 10 degrees radius around the center of the gaze. 4*^th^* & 5*^th^* columns Gaze tracking example while looking downwards: the system uses the whole eye region (shading of the eyelids, shape of the eyelashes, etc) to compute the gaze direction.

The accuracy of the WearCam was assessed in [35] with a group of 10 typically developing children (age 2.4 (0.4) years) and was found to be 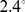 for children and 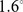 for adult subjects. In typical eye-trackers, gaze direction is computed as a function of geometrical elements such as iris and pupil position, and thus can not be computed when the geometrical elements are occluded. The WearCam does not rely solely on geometrical elements but instead exploits additional features such as the shape and shading of the eyelids and eyelashes. Thus, the system is able to extract information about the gaze direction even when the child is looking downwards and the iris is not completely visible (see Figure 2).

A comparison to other eye-trackers is available in [35]. The accuracy of the WearCam is comparable to the state of the art in eye-tracking technologies, but trades some angular accuracy to be able to cover a much larger field of view. To provide one measure for comparison, the average error of the Tobii T60 with adult subjects using a head-stand is 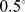 over 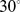, which correspond to an error of 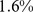 of its field of view. The average error of the WearCam with adult subjects is 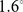 over 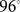, which correspond to an error of 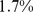 of its field of view (the effective accuracy of the Tobii T60 with young subjects and no head-stand is not available for comparison).

The WearCam uses an offline calibration procedure (described in the *Data Analysis* section) which does not require an active participation of the child. This is done to avoid biases that might incur with children during the calibration of typical eye-tracking devices, such as children not looking at the necessary locations, or gazing elsewhere during the calibration process. For this reasons, the results obtained with the WearCam have a consistent accuracy with all subjects, irrespective of their diagnosis. The only element that is visible by the child when wearing the device is the 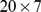 mm mirror, and its impact on the behavior of the child is minimal. In our recordings, while some children looked at the mirror in the initial phase of the recording, they quickly forgot about the device and did not look at the mirror during the protocol.

It should be noted that, as the device is fastened to the head of the child, its measurements are not affected by the movements of the child. This reduces biases that might come from atypical body motions from the children in the ASD group.

### Procedure

**Figure 3:**
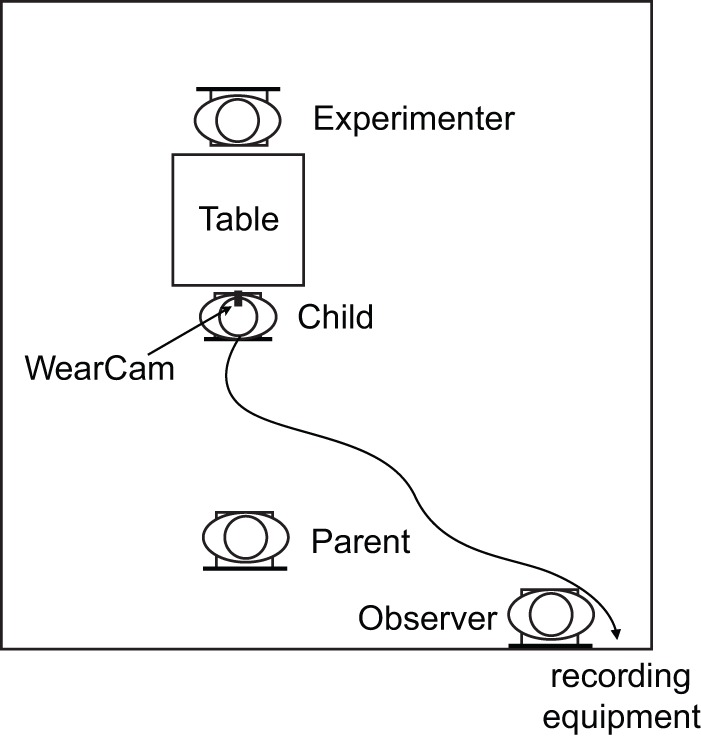
Protocol setup for the experiments.

The experimental protocol comprised four items selected from the abridged version of the ESCS (the ESCS clinical test is a 20-minute videotaped structured observation that enables assessment of a child's initiation and response to nonverbal communication acts (joint attention, social interaction behaviors, requesting behaviors). The ECSC is administered routinely at the CHUV/HUG during clinical screening of ASD in nonverbal children.) [38]. The first item was a soap bubbles blowing game (Object Spectacle Task); followed by playing with a wind-up mechanical toy (Object Spectacle Task); the third item was playing with a small ball (Turn Taking Task) and finally playing with a toy car (Turn Taking Task). The protocol administration lasted in all cases between 5 and 10 minutes and was administered in a naturally lit room. The child was sitting at a table on a child-sized chair, while the experimenter administering the protocol sat at the opposite side of the table also on a low chair. Figure 3 shows a schematic representation of the experimental setup.

The experimenter presented the items and interacted with the child. At all times, the people present in the room consisted of the child, the experimenter, a silent observer and a parent. The parent was placed behind the child and did not interact with her for the duration of the experiment. The observer also was placed behind the child at a distance of several meters so as to minimize the interference on the child's attention. As the WearCam required no calibration, the experiment started as soon as the WearCam had been fastened to the child's head and the mirror aligned so that the child's eyes were clearly visible in the camera's image (fastening and aligning the device takes at most 30 seconds). In a few instances, the camera moved on the head of the child during the experiment (5 instances out of 20 recordings). When that happened, the observer would use a remote control to realign the mirror with the eyes of the child. These occurrences did not interrupt the experiments and did not distract the child. The offline calibration method allowed to ensure that the eye-tracking accuracy was maintained before and after the realignment (typical eye-tracking devices would have required a new calibration phase to be conducted mid-experiment).

#### Data Analysis

The complete interaction was recorded by the WearCam, from the beginning of the interaction to the moment we took off the device after the protocol had ended. We then trimmed the beginning and end of the recording to correspond to the beginning and end of the protocol administration. On average we obtained 6.9 (2.2) (values displayed as Mean (SD)) min of video data per child (ASD: 6.9 (2.2) min, TD: 6.8 (2.3) min). To analyze this data, we used a set of automatic algorithms for tracking gaze and face.

The gaze direction was estimated by analyzing the image of the eyes recorded by the WearCam mirror. Technical information on how this information is extracted can be found in [35]. For each recording, a trained experimenter visualized the video of the field of view and of the eyes in a custom-made software. The experimenter used all identifiable instances in which the direction of the child's gaze was unambiguous (e.g. when the child reached toward an object and the eyes shifted toward it), and placed a calibration point at the corresponding position in the image. This is possible as the eyes of the child are constantly visible in the recorded mirror. The experimenter continued providing additional calibration points until 50 samples were collected. This process lasted 10–15 minutes per video. The experimenters had all worked with the same system in the past and were all familiar with the rating process.

Face detection was accomplished using a semi-automatic method: we began by running an automatic face detection algorithm [43] and then recruited trained human raters (graduate students) who controlled and approved each detection and also indicated faces that were not detected by the automatic system. This semi-automatic system thus ensured that all faces in the video were detected correctly, while lessening the burden of manual labelling. The face labelling process for a single video takes approximately 10 minutes.

After all of the experiments were conducted, three trained raters collected calibration samples for the gaze tracking. Raters then performed the semi-automatic tracking of faces throughout the videos. The raters were blind to the goals of the study and to the diagnosis of the participants. Inter-rater reliability was computed over 40 minutes of video that were labelled by all raters, and showed a correlation 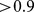. To maintain consistency across experiments, each recording was split into multiple parts corresponding to each item presented by the psychologist, which resulted in item-subsets of durations ranging from 1 to 3 minutes.

We computed the position of the face of the experimenter at any given time, and defined the following measurement variables:

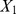
: Proportion of time a face appeared inside the child's field of view (*In FoV*).

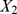
: Proportion of time a face appeared inside the child's Central Vision (*In CV*).

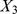
: Frequency of episodes of gaze directed towards a Face (*Episode Frequency*).

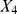
: Duration of episodes of gaze directed towards a Face (*Episode Duration*).

**Figure 4:**
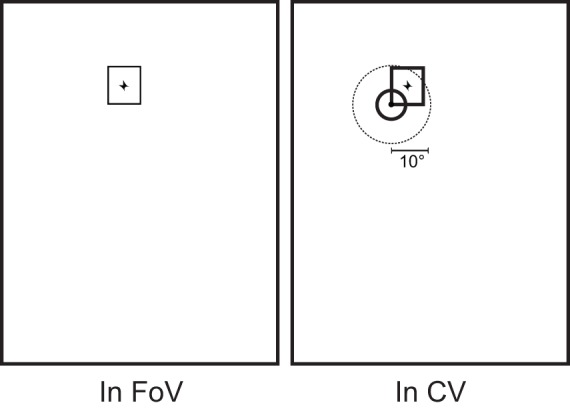
Schema of the events recorded. Whenever a face appeared in a frame, one or more of these events occurred. *in FoV*: a face (rectangle) is present in the broad field of view; *in CV*: a face is inside a 10^°^ radius of the Central Vision (crosshair).

Central Vision (CV) was defined as a circle of 10 deg (radius) around the gaze point, corresponding to foveal and para-foveal vision (see Figure 4 for a schematic representation). 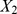 was normalized by the amount of time a face appeared in the field of view. A *Gaze Episode* was defined as the span of time between the instant (image frame) the gaze moved on a face (Face in CV) and the instant it left the face; an episode was marked when this interval was at least 120 ms long (equivalent to 3 frames) to avoid counting short fixations and movements that crossed the face but did not linger there. Gaze episodes were used to avoid the drawbacks related to the explicit computation of fixations (see [44] for a thorough discussion of this issue).

Additionally, we collected the trajectories of gaze for all the recordings and combined the coordinates of gaze from each group to obtain two histograms of the gaze direction throughout the experiments. We then defined the following measurement variables.

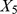
: mean vertical angle of gaze (*Vertical Mean*).

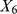
: mean vertical dispersion of gaze (*Vertical Exploration*).

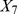
: mean lateral angle of gaze (*Lateral Mean*).

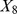
: mean lateral dispersion of gaze (*Lateral Exploration*).

where dispersion was computed as the standard deviation of the gaze distribution. We differentiated the analysis of gaze trajectories to the instances in which the child was looking at the face of the experimenter (with measurement variables 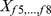), and, conversely when the child was looking elsewhere in the environment (with measurement variables 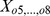). We did not discriminate between looking at particular objects or looking around in the room.

A mixed design 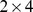 ANCOVA test was run independently for 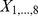 with between-subject factor *Diagnosis* ({ASD, TD}), within-subject factor protocol *Item* ({bubbles, mouse, car, ball}) and covariate *Developmental Age* (years, 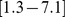). To control for fringe effects of chronological age which might have affected the measurements 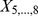, we also performed an additional ANCOVA test, in which we replaced the covariate *Developmental Age* with *Chronological Age*(years, 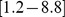). We verified the gaussianity of the distribution of all measurements using a Kolmogorov-Smirnoff test, and ran student t-tests on each measured variable accounting for the Diagnosis factor.

## Results

We present the results of our analysis in two separate sections, focusing on the two different aspects of visual behavior we analyzed. First we describe our analysis of episodes of gaze toward social stimuli, and then more generally to the study of gaze patterns across the whole field of view. A detailed summary of the results is provided in Tables 2, 3 and 4.

**Table 2:** Comparison of gaze factors for TD and ASD groups.

Variable	TD group*^a^*	ASD group*^a^*	T-Tests*^b^*
In Fov	65.28%±26.13	63.07%±24.75	p: 0.673 (DF: 79)
**In CV**	**11.82%**±**10.50**	**7.22%**±**8.63**	**p: 0.022 (DF: 79)**
Episode Frequency	6.75±5.13	4.93±4.90	p: 0.081 (DF: 79)
**Episode Duration**	**0.62**±**0.31**	**0.48**±**0.29**	**p: 0.040 (DF: 79)**
gaze directed to faces			
Lateral Mean	2.64^°^±14.95^°^	0.05^°^±12.63^°^	p: 0.370 (DF: 77)
Vertical Mean	16.97^°^±9.67^°^	19.41^°^±11.34^°^	p: 0.267 (DF: 77)
Lateral Exploration	8.78^°^±5.09^°^	9.29^°^±6.86^°^	p: 0.683 (DF: 77)
Vertical Exploration	6.25^°^±3.61^°^	6.30^°^±4.50^°^	p: 0.949 (DF: 77)
gaze directed to objects			
Lateral Mean	2.96^°^±8.21^°^	1.33^°^±10.45^°^	p: 0.290 (DF: 79)
**Vertical Mean**	9.49^°^±12.33^°^	−0.58±9.94^°^	**p: 0.000 (DF: 79)**
**Lateral Exploration**	9.74^°^±3.35^°^	13.06^°^±4.92^°^	**p: 0.000 (DF: 79)**
Vertical Exploration	13.59^°^±4.10^°^	14.54^°^±5.05^°^	p: 0.316 (DF: 79)

Refer to text for a detailed description of each factor.

* lines in bold present significant differences.

**Table 3:** 2-way ANCOVAs on the variables *In CV* and *Episode Duration*, controlling for Developmental Age.

Source	Sum Sq.	d.f.	Mean Sq.	F	P
In CV					
**Diagnosis**	0.03	1	0.03	**4.17**	**0.046**
**Item**	0.16	3	0.05	**7.09**	**0.000**
DevAge	0.02	1	0.02	2.80	0.100
Interactions					
Diagnosis*Item	0.01	3	0.00	0.28	0.839
Diagnosis*DevAge	0.01	1	0.01	1.49	0.228
Item*DevAge	0.01	3	0.00	0.43	0.734
Error	0.39	52	0.01		
Total	0.64	67			
Episode Duration					
**Diagnosis**	0.57	1	0.57	**7.13**	**0.010**
Item	0.18	3	0.06	0.75	0.525
DevAge	0.17	1	0.17	2.12	0.151
Interactions					
Diagnosis*DevAge	0.01	1	0.01	0.14	0.707
Diagnosis*Item	0.14	3	0.05	0.58	0.629
Item*DevAge	0.17	3	0.06	0.70	0.554
Error	4.17	52	0.08		
Total	5.50	67			

* lines in bold correspond to significant effects.

**Table 4:** 2-way ANCOVAs on the variables *Mean Elevation* and *Lateral Exploration*.

Controlling for Dev. Age					
Source	Sum Sq.	d.f.	Mean Sq.	F	P
Vertical Mean (objects)					
**Diagnosis**	1587.85	1	1587.85	**15.21**	**0.000**
**Item**	2725.92	3	908.64	**8.70**	**0.000**
DevAge	75.99	1	75.99	0.73	0.398
Interactions					
Diagnosis*Item	156.01	3	52.00	0.50	0.685
Diagnosis* DevAge	4.31	1	4.31	0.04	0.840
Item*DevAge	189.74	3	63.25	0.61	0.614
Error	5430.07	64	104.42		
Total	10274.58	79			
Lateral Exploration (objects)					
**Diagnosis**	239.86	1	239.86	**18.60**	**0.000**
Item	57.91	3	19.30	1.50	0.226
**DevAge**	86.60	1	86.60	**6.71**	**0.012**
Interactions					
Diagnosis*Item	16.86	3	5.62	0.44	0.728
Diagnosis* DevAge	15.89	1	15.89	1.23	0.272
Item*DevAge	62.72	3	20.91	1.62	0.196
Error	670.73	64	12.90		
Total	1169.70	79			

Controlling for Chron. Age					
Source	Sum Sq.	d.f.	Mean Sq.	F	P
Vertical Mean (objects)					
**Diagnosis**	1587.85	1	1587.85	**14.83**	**0.000**
**Item**	2757.80	3	919.27	**8.59**	**0.000**
ChrAge	49.74	1	49.74	0.46	0.499
Interactions					
Diagnosis*Item	155.42	3	51.81	0.48	0.695
Diagnosis* ChrAge	1.42	1	1.42	0.01	0.909
Item*ChrAge	66.48	3	22.16	0.21	0.891
Error	5567.34	64	107.06		
Total	10274.58	79			
Lateral Exploration (objects)					
**Diagnosis**	239.86	1	239.86	**16.02**	**0.000**
Item	53.08	3	17.69	1.18	0.326
ChrAge	29.26	1	29.26	1.95	0.168
Interactions					
Diagnosis*Item	23.09	3	7.70	0.51	0.674
Diagnosis* ChrAge	0.29	1	0.29	0.02	0.890
Item*ChrAge	36.57	3	12.19	0.81	0.492
Error	778.54	64	14.97		
Total	1169.70	79			

* lines in bold correspond to significant effects.

### Gaze episodes to faces

**Figure 5:**
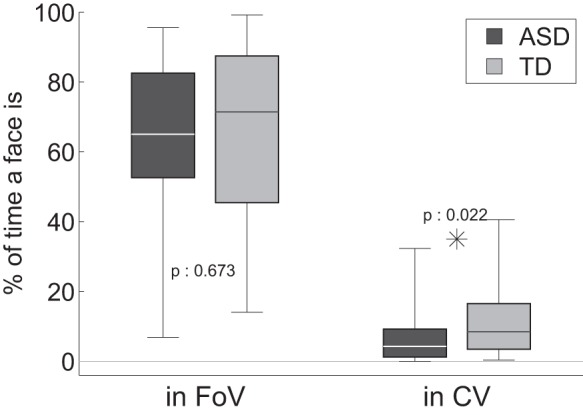
Analysis of gaze directed toward faces. *in FoV*: Percentage of time a face was in the broad field of view. *in CV*: Percentage of time a face was in central vision.

**Figure 6:**
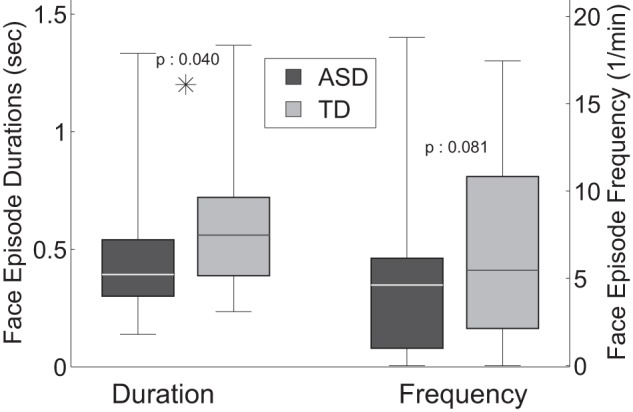
Duration and frequency of episodes of gaze directed toward a face.

We begin with the results on the analysis of gaze episodes directed towards the face of the experimenter. Both groups kept the face of the experimenter within their field of view (*In FoV*) for comparable amounts of time (ASD: 63.1%
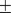
24.8%, TD: 65.3%
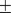
26.1%, p:0.673). This suggests that both groups were orienting towards the experimenter for the same amount of time (see Figure 5). Children in the ASD group, however, kept the face of the experimenter inside their Central Vision (*In CV*) significantly less than children in the TD group (ASD: 7.2%
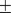
8.6%, TD: 11.8%
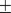
10.5%, p:0.022). When children with ASD looked at the face of the experimenter, they did so for shorter lapses of time (*Episode Duration*) (ASD: 0.48
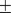
0.29 sec, TD: 0.62
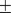
0.31 sec, p:0.040) (see Figure 6).

When studying the effects and interactions of the *Diagnosis* and *Item* factors, and controlling for the effect of *Developmental Age* (see Table 3), we found no main effects or interactions on the 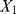 (*In FoV*) variable. We measured, however, a main effect on the 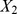 (*In CV*) variable for *Diagnosis* (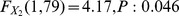) and *Item* (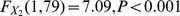), with no interaction between factors. The effect of item is not surprising, as different tasks may elicit different types of gaze behavior (e.g. turn taking: 7.3% (4.7%) vs. object spectacle tasks 5.4% (3.7%) for all children). However, as children from the two groups played each item for comparable amounts of time (for ASD, Bubbles: 65.1 (55.6) sec, Mouse: 193.7 (101.1) sec, Car: 96.3 (61.7) sec, Ball: 52.2 (27.7) sec; for TD, Bubbles: 63.2 (28.7) sec, Mouse: 145.5 (60.8) sec, Car: 105.4 (70.3) sec, Ball: 57.7 (22.3) sec), the results do not seem to be biased by the experimental protocol. Finally, we found a main effect for *Diagnosis* on the 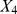 (*Episode Duration*) variables (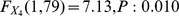) with no interactions.

### Gaze patterns across the field of view

We now describe the results of our analysis of gaze patterns across the field of view. When the children were looking at the face of the experimenter, we found no significant differences in the gaze patterns. We will therefore focus on the gaze patterns when children were looking at objects rather than the face of the experimenter.

**Figure 7:**
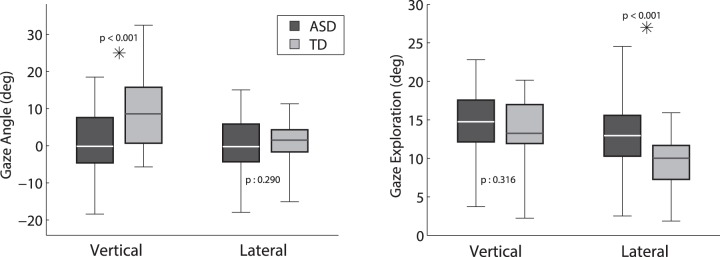
Analysis of gaze dispersion across the field of view. Mean vertical and lateral angles of the gaze when children were looking at non-social stimuli *(top)* and exploration of the gaze in the vertical and lateral directions *(bottom)*.

The mean elevation angle (*Vertical Mean*) for the ASD group was 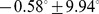 and for the TD group 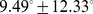 (see Figure 7). The difference between the two groups is very significant (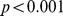) and suggests that children in the ASD group tended to look slightly downwards compared to the TD group. While the lateral angle on average was not significantly different between the two groups, the variance of the lateral angle (*Lateral Exploration*) was significantly larger (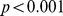) for children in the ASD group (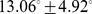) than in the TD group (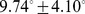).

**Figure 8:**
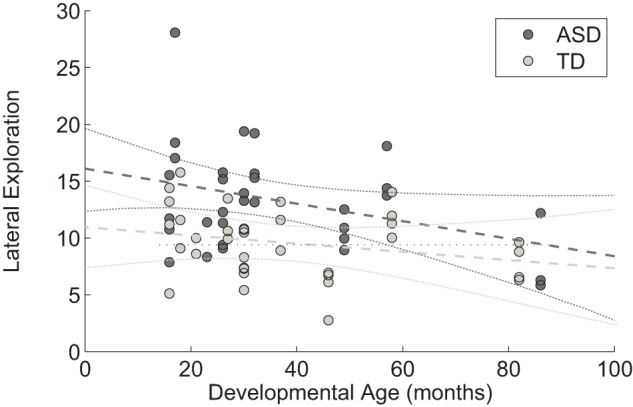
Lateral exploration as a function of developmental age. For each child, the results of the 4 protocol items are displayed separately.

The mixed design ANCOVA test (see Table 4) found a main effect on 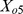 (*Vertical Mean*) for *Diagnosis* (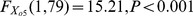) and *Item* (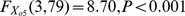) with no interactions. The effect of *Item* is likely due to the fact that some tasks required the child to look higher than others (e.g. Blowing bubbles: 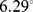 (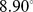) vs. Toy car: 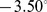 (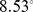) for all children). We found a main effect on 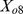 (*Lateral Exploration*) for *Diagnosis* (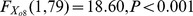) and *DevAge* (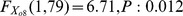), with no interaction. The results suggest that ASD children tended to make more extensive use of their lateral field of view than the TD group. However, developmental age seems to also play a role in the amount of lateral exploration (as can be seen in Figure 8), indeed, younger children display a higher exploration of the lateral field of view. When controlling for *Chronological Age* instead of *Developmental Age* we found similar effects of *Diagnosis* for 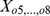, but found no effects or interactions for *Chronological Age*.

### Reliability and Limitations

A number of elements might impact the reliability of the results presented. Firstly, the availability of subjects was a factor in the selection of the control group, and the study would have benefitted from a control population matching in both chronological and developmental age. Nevertheless, the development of central and peripheral vision has been shown to be fully developed by month 13 in typically developing children [45]. Moreover, the perception and reaction to social stimuli such as eye contact and joint attention cues is also present by the first year of life [25]. Therefore, the bias induced from having a (chronologically) younger control population should not be significant. Indeed, our results show no statistical effect of chronological age on the variables we measured, which suggests that this factor did not play a negative role on the experiment.

It must also be noted that the number of samples for this study was relatively low (20 children, with 4 measurements per child) for a 2x4 ANCOVA test with one covariate. However, the absence of interactions between factors, and between factors and covariate, suggests that the statistics are sufficient to provide a reliable analysis of the results we obtained.

## Discussion

This study investigated gaze strategies of children with ASD when engaged with a familiar adult in a semi-naturalistic dyadic interaction. Our results show that children with ASD looked significantly less and for shorter amounts of time at the face of the adult interacting with them than their TD counterparts. This difference is of special interest when we take into account the fact that both ASD and TD groups kept the face of the adult inside their broad field of view for comparable amounts of time. Moreover, when looking more generally at the environment, ASD children directed their gaze further down and explored their lateral field of view more extensively than TD children.

### Gaze strategy to human faces

Our result are congruent with other reports of a lower tendency to gaze at faces in children with ASD. Early on, studying children with ASD in free play, two studies [46], [47] noticed that these children tended to turn their gaze away from the adult they were interacting with more frequently than a control group. Other more recent studies present similar results. Swettenham et al. [22] noted that ASD children spend less time focusing on faces in free play than TD children and when they do focus, they do so for a shorter time than their TD counterparts. ASD children spent more time looking at toys. Klin et al. [11] studied how ASD adults watched videos featuring people or objects in a social setting. The ASD adults spend more time watching objects and when they do look at faces, their gaze settles around the mouth instead of the eyes.

Various studies have come up with explanations as to why ASD subjects do not focus their gaze on faces. Trepagnier and colleagues, and Pelphrey and colleagues [7], [9] suggest that ASD subjects have trouble processing faces on a neuronal level, and thus do not find faces as stimulating as TD children do. This could explain why ASD children focus less on faces even when still quite young. As they grow and lack experience looking at faces, they find it hard to recognize facial expressions; this in turn, makes it hard for ASD adults to analyze emotions (see [8] for a review). However, the empirical bases for a deficit in the processing of faces are somewhat controversial [48]. Another element that might come into play is the difficulty ASD children have in switching their attention from one task or stimulus towards another. Swettenham et al. [22] noted that at the age of 2, ASD children already found it harder than TD children to switch their attention from an object towards a person. Studying the shifting of visual attention from non-social stimuli, Landry and Bryson [49] and Elison and colleagues [50] remarked a systematic delay in the reaction times of ASD children. It is not surprising therefore that ASD children spent more time looking at objects than TD children do.

### Gaze toward specific facial features

In our study, we measured the instances of gaze directed to the whole face of the experimenter. Indeed, in our recordings it was not possible to discriminate whether the gaze was directed more toward the eyes or more toward the mouth (or any other facial feature). This is due to a technical limitation of the eye-tracking equipment we used. The Wearcam provides an accuracy of 2.4 degrees over the whole field of view [35]. To be able to distinguish across facial regions scanned by the child's eyes would have required the child to sit about 50 to 75 cm away from the experimenter. While this may be difficult to ensure practically during live ecological settings, this would also create a rather odd situation. Indeed, such interpersonal distance may be qualified as intimate. Little is known of what effect such intimate settings have on children with ASD. Pedersen and Schelde [51] reported large individual difference in ASD children as to what would be deemed a comfortable interpersonal distances. They found a distance of 0 to 50 cm to be preferred by children with autism affected by severe mental retardation, while a distance of 50 cm to 1.5 m was preferred by less affected ASD children. Kennedy et al. [52] indicate that the perception of personal space may be regulated by the amygdala. Both accounts are consistent with the reported atypical functioning of the amygdala [10], [53]. To avoid introducing a bias due to interpersonal distance, we preferred the standard set-up used in the ESCS tests.

### Interaction in a natural environment

It is not always easy to elicit atypical behavior in a structured experiment. Often gaze peculiarities of individuals with ASD “[are] not readily apparent, especially in controlled laboratory tests.” [2]. One would hence prefer video display of social scenes to static images [6]. Better even would be to monitor visual behavior in a live interaction either through video-based display [28], [54] or in a true ecological setting, similarly to what we did in our study.

We opted for a naturalistic situation where the child engaged in a dyadic interaction with an adult partner. Child and adult were physically immersed in the environment in which the interaction took place. The child was let free to engage in reciprocal interaction. Through the use of items from the ESCS that monitor for both a proactive and a reactive attitude to engaging in joint attention tasks, the child was given the opportunity to not only respond but also initiate the interaction, in a way that is close to naturalistic play [39]. Such bilateral interaction are fundamental to human social interactions and it was thus interesting to monitor gaze toward the adult in both settings. Competence for such contingent exchange are a crucial component to the development of communication in children and are present early in development in typically developing children [54]. We hypothesized that by offering the children such a direct contact with the interaction partner – as opposed to doing it via a video display as we did in previous work [55]– we would elicit a more natural and unbiased gaze behavior both from the ASD and TD children.

Studies of ASD children gaze behavior in ecological settings are scarce. Structured experimental protocols are often preferred because of their repeatability but also because nowadays a large battery of technological tools allow one to rapidly and systematically analyze the data via dedicated software. In contrast analyzing data from experiments conducted in ecological settings usually require a very tedious manual labeling of the video recordings of the interaction. The labeling for these types of study had to be performed by at least two raters to avoid subjective interpretation of the scene. However, since one could not explicitly reconstruct where the child was looking, one would constrain the environment or the interaction in such a way as to avoid any ambiguity and one would mostly rely on head motion as an indicator of eye direction. The very recent advances made in wearable eye tracking technology, which we exploit here, will reduce these technological difficulties. In particular, by providing a first-person view, wearable eye-trackers offer a reliable measurement of where and what the child is looking at. Increased use of these systems will, in the years to come, allow tremendous advances in our understanding of how children with ASD perceive the world in their daily routines.

### Lateral gaze, eccentric viewing and peripheral vision

Our data revealed an increased lateral exploration of the visual field and a marked preference for looking down in children with ASD. These particularities do not seem to be related solely to a lack of interest to social stimuli. Indeed, children with ASD kept the adult in their field of vision just as much as their TD counterparts. Thus, there are other hypotheses that may help explain our results.

#### Downcast gaze

The phenomenon of downcast gaze is a well known symptom of autism (see [2] for a review). Bogdashina [56] links the downcast gaze to a sensorial overload coming from a hypersensitivity to visual stimuli. The reasons for this hypersensitivity would be an “inability to filter excessive or irrelevant information”, a “distorted perception” that brings anxiety, confusion and stress. By looking downwards, these children very likely look at static stimuli (ground, table), that are less susceptible to perturb them. Indeed in our experiments, most visual stimuli appeared in the upper field of vision (e.g. the experimenter, windows). A hypersensitivity to these stimuli would explain the gaze directed downwards. This hypothesis is coherent with the theory of Enhanced Perceptual Functioning (EPF) [19] which suggests that ASD children are overly sensitive to high frequency visual signals and proposes the use of an eccentric viewing strategy as a way to filter these signals.

#### Lateral gaze and eccentric viewing

In a study of visual exploration of objects, Mottron et al. [23] found that ADS children used eccentric viewing, and more precisely episodes of “lateral glances”, as a strategy to “regulate the amount of local information in [a] scene”. Indeed, one sees less details when directing the eyes sideways. By looking laterally we thus apply a low-pass filter of visual stimuli, which reduces the high frequency signals. This allows to explain the well known symptom of looking at someone “out of the corner of the eyes” [57], [58].

However, the use of lateral glances does not explain entirely the extended lateral exploration we measured in children with autism. This strategy of eccentric vision may not be restricted to specific episodes of lateral glances. It may be that this filtering strategy is present in the gaze patterns across the whole visual field and not solely in lateral glances. Such a strategy is difficult to measure as it is less explicit than the instances of lateral glances. We are not aware of any study that has tried to validate this hypothesis. Although we did not measure this phenomenon when children were looking at faces, the striking differences we found when restricting the analysis to non-social stimuli suggest that this could be an interesting direction for further research.

#### Local vs. Global features

In a monitor-based eye-tracking study, Shic et al. [59] studied the gaze patterns of ASD children looking at naturalistic images. They showed that children with autism had a preference for local features, and were less affected by perturbations of the images such as scene inversion. Moreover, they showed that children with ASD used less motion information, which is consistent with motion processing deficits reported in the literature (e.g. [60]). The preference of children with autism for local features could explain why children in the ASD group used their lateral field of view more extensively, as they would need to examine directly local features of objects and the environment more than the control children.

A further analysis of the recordings, extracting motion and local contrasts as well as measuring the child's head motion, could bring to light more differences in the use of low-level features in autism, and will likely be the focus of future studies.
